# Crossing double stent retriever technique for refractory terminal internal carotid artery occlusion

**DOI:** 10.1016/j.radcr.2022.03.023

**Published:** 2022-04-04

**Authors:** Isao Sasaki, Taichiro Imahori, Tatsuya Yano, Masanori Gomi, Junko Kuroda, Norikata Kobayashi, Kimitoshi Sato, Yoji Niwa, Koichi IwasaKi, Hiroshi Hasegawa

**Affiliations:** a Department of Neurosurgery, Ainomiyako Neurosurgery Hospital, Osaka, Japan; b Department of Neurosurgery, Kobe University Graduate School of Medicine, 7-5-2, Kusunoki-cho, Chuo-ku, Kobe City, Hyogo 650-0017, Japan

**Keywords:** Acute ischemic stroke, Large vessel occlusion, Endovascular treatment, Mechanical thrombectomy, Stent retriever, Device-clot-vessel interaction, MT, mechanical thrombectomy, SR, stent retriever, ICA, internal carotid artery, MRI, magnetic resonance imaging, ACA, anterior cerebral artery

## Abstract

Mechanical thrombectomy is highly effective for the recovery of acute ischemic stroke with large vessel occlusion. However, refractory occlusions are still encountered despite the use of currently available devices. In this article, we present a case of refractory terminal internal carotid artery occlusion treated with the “crossing double stent retriever technique.” Two thrombectomy procedures with the combined technique using a stent retriever and aspiration catheter failed to recanalize the terminal internal carotid artery occlusion that involved the dominant anterior cerebral artery. We then applied the crossing double stent retriever technique as a rescue technique. Two microcatheters were advanced across the occlusion: one to the anterior cerebral artery and the other to the middle cerebral artery. First, a Trevo NXT 4 mm stent retriever was deployed from the anterior cerebral artery. Next, an additional Trevo NXT 4 mm stent retriever was deployed from the middle cerebral artery, and full immediate restoration of flow was achieved on angiography. Intraprocedural radiological images showed that the 2 microcatheters traversed different pathways, and the 2 stent retrievers completely covered the entire vessel with apparent in-stent clot sign. Both stent retrievers were then pulled back together, and a hard clot was retrieved. Subsequent angiography revealed complete recanalization. The crossing double stent retriever technique seems an effective rescue technique for treating refractory terminal internal carotid artery occlusion, especially with the anatomical feature of branching of the dominant anterior cerebral artery. This technique can facilitate the device-clot-vessel interaction by engaging the clot via 2 different device pathways.

## Introduction

Endovascular recanalization by mechanical thrombectomy (MT) is highly effective for the recovery of patients with acute ischemic stroke who have large vessel occlusion [1]. However, refractory occlusions are still encountered despite the use of currently available thrombectomy devices [2]. Recently, the “double stent retriever (SR) technique,” in which 2 SRs are used simultaneously at the occlusion site, has been reported as a rescue thrombectomy technique [3], [4], [5], [6], [7], [8], [9], [10], [11]. Previously, we reported that adding one more SR in the same axis at the occlusion facilitates the device-clot interaction, which can be recognized as improved stent coverage in radiographic images during device deployment [11]. In addition, to address the impact of complicated vascular anatomy, it is necessary to gain insight into the mechanism underlying the effectiveness of the procedure, taking into account the “device-clot-vessel interaction” [12,13].

In this report, we present a case of refractory terminal internal carotid artery (ICA) occlusion treated with the “crossing double SR technique,” which is a method of intentionally changing the course of the devices across the clot to better match the vascular geometry. 

## Case presentation

**Fig. 1:**
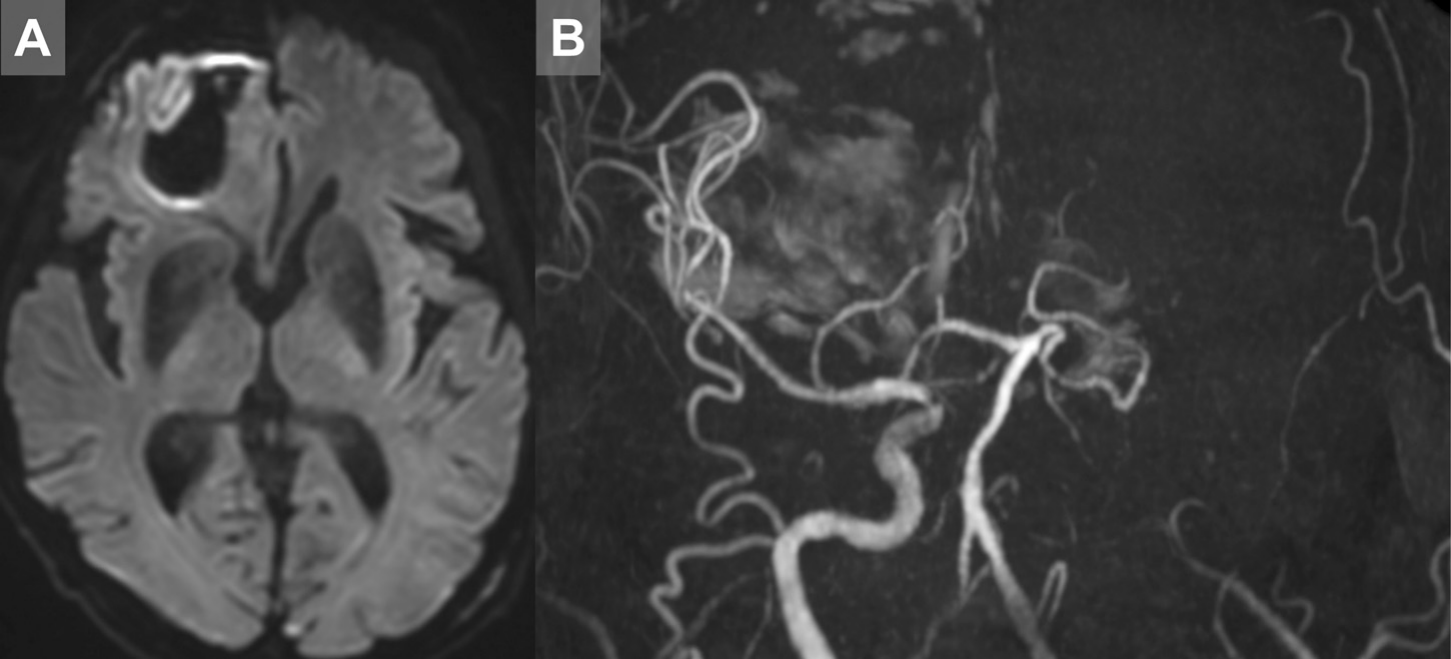
Preprocedural image (A and B) Emergency magnetic resonance imaging showing slight acute ischemic changes and occlusion of the left internal carotid artery. The bilateral anterior cerebral arteries were not visualized.

An 87-year-old man with a history of chronic heart failure was admitted to our institute because of trauma. The patient suddenly developed reduced consciousness with a National Institutes of Health Stroke Scale score of 26. Magnetic resonance imaging (MRI) revealed occlusion of the left ICA with slight acute ischemic changes (Fig. 1A, B). The bilateral anterior cerebral arteries (ACAs) were also not observed. Emergency endovascular treatment was performed without intravenous thrombolysis because of the presence of traumatic intracranial hemorrhage. Written informed consent was obtained from the patient's family member before the procedure.

**Fig. 2:**
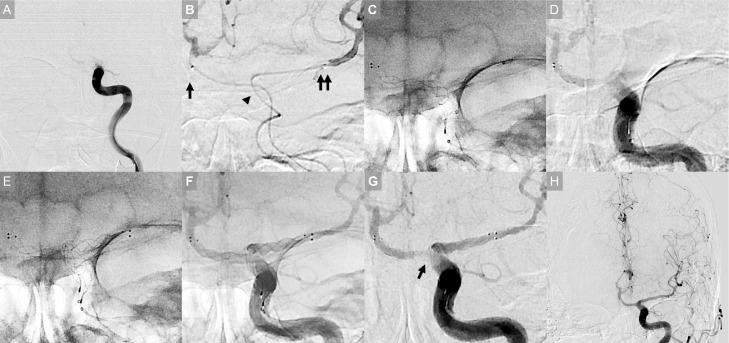
Intraprocedural image (A) Initial angiography showing occlusion of the left terminal internal carotid artery (ICA). (B) After 2 unsuccessful thrombectomy procedures, we applied the “crossing double SR technique.” Two microcatheters were advanced across the occlusion, one to A1 of the anterior cerebral artery (arrow) and the other to the distal M1 of the middle cerebral artery (double arrow). Note the apparent difference in the course of the 2 microcatheters at the occlusion site (arrowhead). (C) A Trevo NXT 4 × 28 mm stent retriever (SR) was deployed from the A1 to ICA. (D) Angiography showing partial immediate restoration of flow. (E) One more Trevo NXT 4 × 28 mm SR was deployed from the M1 to ICA (F) Angiography showing full immediate restoration of flow. Native angiography image showing that the 2 SRs in total fully covered the inside of the vessel. (G) Digital subtraction angiography image showing the “in-stent clot sign,” which is the filling defect of the clot in the strut and the sign indicating the device-clot interaction (arrow). Thereafter, both deployed SRs were pulled back together, and a hard clot was retrieved. (H) Subsequent angiography revealing complete recanalization.

Initial angiography revealed occlusion of the left terminal ICA (Fig. 2A). The microcatheter tended to enter A1 of the ACA, and it was challenging to be directed to M1 of the middle cerebral artery. Despite the considerable difficulty, the microcatheter was placed at the distal M1. However, 2 passes of MT procedures with the combined technique using both an SR and aspiration catheter failed to recanalize the occlusion and did not retrieve any piece of the clot.

We then applied the “crossing double SR technique” as a rescue thrombectomy technique during the third pass. Two microcatheters were advanced across the occlusion, one to A1 and the other to the distal M1 (Fig. 2B). First, a Trevo NXT 4 × 28 mm SR (Stryker, Kalamazoo, MI, USA) was deployed from A1 to ICA, and partial immediate restoration of flow was confirmed on angiography (Fig. 2C, D). Next, an additional Trevo NXT 4 × 28 mm SR was deployed from M1 to ICA, and full immediate full restoration of flow was noted (Fig. 2E–G). Both deployed SRs were then pulled back together under aspiration through a balloon guide catheter, and a hard clot was retrieved. Subsequent angiography revealed Thrombolysis in Cerebral Infarction 3 recanalization (Fig. 2H). The time from femoral access to recanalization was 79 minutes, and the time from stroke onset to recanalization was 145 minutes.

**Fig. 3:**
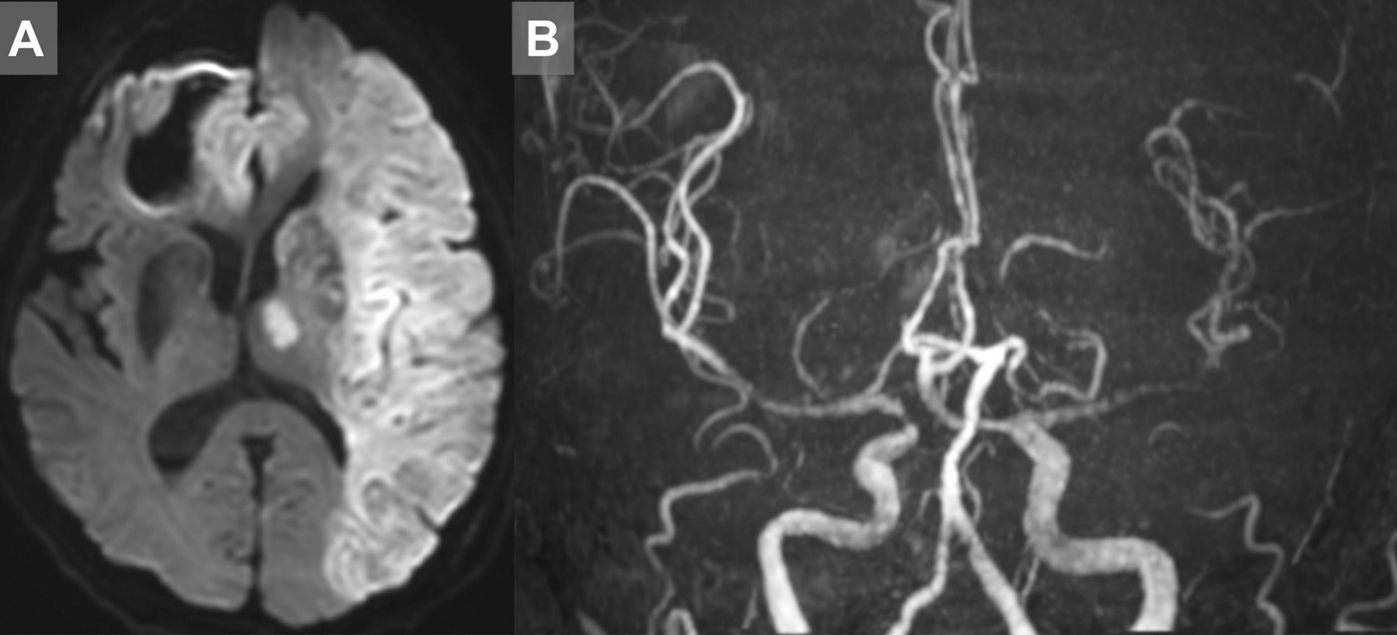
Postprocedural image (A and B) Magnetic resonance imaging performed on the day after the procedure, showing successful revascularization but extensive infarction.

Computed tomography performed after the procedure did not reveal any intracranial hemorrhage associated with the endovascular procedure. The day after the procedure, MRI - showed successful revascularization, but with extensive infarction. (Fig 3A, B). The patient did not recover and died 2 days after the procedure.

## Discussion

We reported a case of refractory terminal ICA occlusion that was treated using the crossing double SR technique. This technique seems effective for treating refractory terminal ICA occlusion, especially with the anatomical feature of dominant ACA branching. This technique can facilitate device-clot-vessel interaction- by engaging the clot via 2 different device pathways.

Our current case suggests that the crossing double SR technique is an effective rescue technique to treat refractory terminal ICA occlusion involving the dominant ACA. Although recanalization was achieved in this case, it did not benefit the patient because of the time delay. However, this case suggests that special consideration may be required for terminal ICA occlusion involving the dominant ACA. The following pitfalls can exist with such vascular geometry: difficulty navigating the microcatheter into the middle cerebral artery, low impact of the thrombectomy device on the clot if used from M1, and limited cerebral ischemic tolerance [14], [15], [16]. In such cases, it should be noted to take effective rescue measures at an early stage that are different from the conventional strategies.

**Fig. 4:**
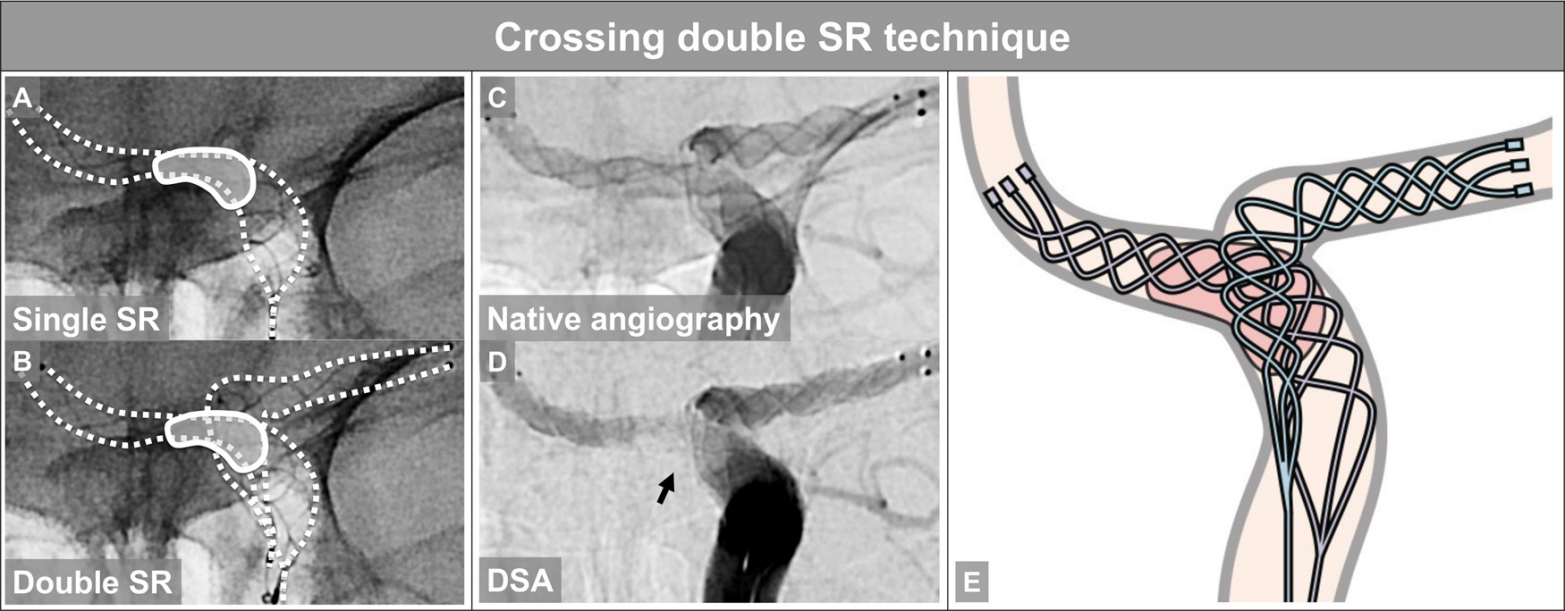
Crossing double SR technique (A) Single stent retriever (SR). The dotted line indicates the area covered by the SR, and the solid line indicates the clot. (B) Double SR. (C) Native angiography image. Note that the 2 SRs notably covered the entire vessel, traversing different pathways. (D) Digital subtraction angiography (DSA) image showing the “in-stent clot sign,” which is the filling defect of the clot in the strut and the sign indicating the device-clot interaction (arrow). (E) Schematic of the crossing double SR technique for a terminal internal carotid artery. This technique can facilitate the device-clot-vessel interaction by intentionally changing the course of the device across the clot to better match the vessel geometry.

The crossing double SR technique can facilitate the device-clot-vessel interaction by intentionally changing the course of the devices across the clot to better match the vessel geometry. It is now well recognized that in addition to the device-clot relationship, vessel morphology also influences the success of MT, a concept referred to as the device-clot-vessel interaction [12,13]. The intraprocedural images in the current case clearly showed the “in-stent clot sign,” which is the filling defect of the clot in the strut and the sign indicating the device-clot interaction according to our previous study (Fig. 4) [17]. The device may more successfully engage the clot in a complex vascular morphology situation by intentionally changing the pathway.

The crossing double SR technique has some potential disadvantages. The main concern is the risk of vascular damage, which may be higher than that associated with the usual single SR use. Clinicians should carefully monitor live angiographic images and device resistance [18], [19], [20]. The potential for breakage of the 2 SRs also exists but may be avoided by a simultaneous slow retraction maneuver through the balloon guide catheter [11]. Another problem related to this technique is the increased cost of using 2 SRs. Therefore, this technique should be used in selected cases of refractory occlusion. Further studies are needed to clarify the situations in which this technique is more beneficial than other methods.

## Conclusions

Our case suggests that the crossing SR technique is an effective rescue technique for treating refractory terminal ICA occlusion, especially with the anatomical feature of dominant ACA branching. This technique can facilitate device-clot-vessel interaction by intentionally changing the course of the devices across the clot to better match the vessel geometry.

## Patient consent

Informed consent has been obtained from the patients family member for publication of the case report and accompanying images.
