# Erratum to “Crossing double stent retriever technique for refractory terminal internal carotid artery occlusion” [Radiology Case Reports 17 (2022) 1848-1852]

**DOI:** 10.1016/j.radcr.2023.12.036

**Published:** 2024-01-12

**Authors:** Isao Sasaki, Taichiro Imahori, Tatsuya Yano, Masanori Gomi, Junko Kuroda, Norikata Kobayashi, Kimitoshi Sato, Yoji Niwa, Koichi IwasaKi, Hiroshi Hasegawa

**Affiliations:** a Department of Neurosurgery, Ainomiyako Neurosurgery Hospital, Osaka, Japan; b Department of Neurosurgery, Kobe University Graduate School of Medicine, 7-5-2, Kusunoki-cho, Chuo-ku, Kobe City, Hyogo 650-0017, Japan

The Publisher would like to inform you that during the publication process the following declaration of competing interests was omitted in error.  

**Declaration of competing interests**

We herein state, that this manuscript has not been published nor submitted elsewhere. Furthermore, all authors have read and approved the manuscript. Finally, there are no financial arrangements or other relationship that could be construed as a conflict of interest.  

The Publisher apologizes for any inconvenience caused by this mistake.

